# A Single Injection of an Optimized Adeno-Associated Viral Vector into Cerebrospinal Fluid Corrects Neurological Disease in a Murine Model of GM1 Gangliosidosis

**DOI:** 10.1089/hum.2018.206

**Published:** 2020-11-13

**Authors:** Christian Hinderer, Brenden Nosratbakhsh, Nathan Katz, James M. Wilson

**Affiliations:** Gene Therapy Program, Department of Medicine, University of Pennsylvania Perelman School of Medicine, Philadelphia, Pennsylvania, USA.

**Keywords:** GM1 gangliosidosis, AAV, CSF, lysosomal storage disease

## Abstract

GM1 gangliosidosis is a rare neurodegenerative lysosomal storage disease caused by loss-of-function mutations in the gene encoding beta-galactosidase (β-gal). There are no approved treatments for GM1 gangliosidosis. Previous studies in animal models have demonstrated that adeno-associated viral (AAV) vector-mediated gene transfer to the brain can restore β-gal expression and prevent the onset of neurological signs. We developed an optimized AAV vector expressing human β-gal and evaluated the efficacy of a single intracerebroventricular injection of this vector into the cerebrospinal fluid (CSF) of a murine disease model. The AAV vector administration into the CSF increased β-gal activity in the brain, reduced neuronal lysosomal storage lesions, prevented the onset of neurological signs and gait abnormalities, and increased survival. These findings demonstrate the potential therapeutic activity of this vector and support its subsequent development for the treatment of GM1 gangliosidosis.

## Introduction

GM1 gangliosidosis is a rare genetic disorder caused by mutations in the *GLB1* gene, which encodes lysosomal beta-galactosidase (β-gal). In the absence of β-gal, cells are unable to catabolize polysaccharides with terminal galactose residues. Keratan sulfate, a galactose-containing glycosaminoglycan, accumulates in a variety of tissues, causing skeletal dysplasia, hepatosplenomegaly, and cardiomyopathy.^1^ In the central nervous system (CNS), the inability to catabolize GM1 ganglioside results in marked neuronal GM1 storage and subsequent neurodegeneration.^1^

The clinical presentation of GM1 gangliosidosis varies dramatically between patients depending on the underlying GLB1 mutations.^2^ Patients expressing residual β-gal may present with neurological signs at any age and variable degrees of somatic disease. In rare cases, patients present with peripheral manifestations of β-gal deficiency without CNS involvement, a disease termed mucopolysaccharidosis type IVB.

The most common form of GM1 gangliosidosis is the infantile subtype, which occurs in patients with little or no residual β-gal expression.^1–3^ Infantile GM1 gangliosidosis is characterized by a uniformly rapid neurodegenerative course beginning in the first 6 months of life, with a survival of less than 5 years.^1,3^

Previous studies in murine and feline models of GM1 gangliosidosis demonstrated that adeno-associated viral (AAV) vectors can be used to deliver the normal *GLB1* coding sequence to neurons, resulting in long-term expression of the β-gal enzyme.^4,5^ β-gal is secreted by transduced cells and can be taken up by surrounding cells via binding to the mannose-6-phosphate receptor, resulting in widespread distribution of the enzyme.^5,6^ This phenomenon allows for the correction of storage lesions throughout the CNS after a small number of vector injections into the brain parenchyma.

Studies in canine and feline models of other lysosomal storage diseases have shown that gene transfer to the brain and spinal cord can alternatively be achieved by using AAV vector delivery into the cerebrospinal fluid (CSF).^7–9^ This approach allows for more widespread gene transfer with a single, minimally invasive injection and appears to carry less risk of local toxicity at the injection site.^7–17^ The AAV delivery into the CSF achieves efficient gene transfer to the brain and spinal cord even in the presence of systemic neutralizing antibodies to the vector capsid, making this approach applicable to patients regardless of the presence of pre-existing AAV antibodies.^9,11,12,14^

The aim of this study was to develop an optimized AAV vector expressing human β-gal and evaluate the impact of vector administration into the CSF on brain enzyme activity, lysosomal storage lesions, and neurological signs and survival by using a murine disease model.

## Materials and Methods

### Animal procedures

All animal procedures were approved by the Institutional Animal Care and Use Committee of the University of Pennsylvania. *Glb1* knockout mice (*Glb1^−/−^*; RBRC00690) were obtained from RIKEN BioResource Research Center. Mice were maintained as heterozygous carriers *Glb1^+/−^* in a C57BL/6J background. Mice were genotyped by PCR from tail snips at 3 weeks of age. For intracerebroventricular (ICV) injections, vectors were diluted in artificial CSF (1 mM phosphate, pH 7.2, 150 mM NaCl, 3 mM KCl, 1.4 mM CaCl_2_, 0.8 mM MgCl_2_, 0.001% Pluronic F-68.) to a volume of 5 μL, and injections were administered freehand on isoflurane-anesthetized mice by using a custom gastight syringe (Hamilton) and a cemented 10 mm 27 gauge needle, with plastic tubing attached to the needle base to limit penetration to a depth of 3 mm. The ICV injections were performed by a board-certified laboratory animal veterinarian. The ICV injection proficiency was verified based on 100% successful lateral ventricle delivery of a reference compound in 100 sequential injections.

Based on the historical rate of successful ICV delivery, the expected frequency of vector delivery outside of the ventricle in this study is <1%. Submandibular blood collection was performed on isoflurane-anesthetized mice. Blood was collected in serum separator tubes, allowed to clot, and separated by centrifugation before aliquoting and freezing at ≤−60°C. Animals were euthanized at the scheduled necropsy time points or if they met one or more of the following euthanasia criteria: ≥20% weight loss from previous weight monitoring, inability to move right from a supine position in less than 15 s, and paralysis of two or more limbs. Determination of the need for euthanasia was made by an evaluator blinded to the treatment group at the time of assessment.

At the time of necropsy, mice were sedated with ketamine and xylazine (intraperitoneal injection, 100/10 mg/kg) and CSF was collected by suboccipital puncture by using a 32 gauge needle connected to polyethylene tubing. Euthanasia was performed by cervical dislocation. CSF, heart, lung, liver, and spleen were immediately frozen on dry ice and stored at ≤ −60°C. Brains were removed, and a coronal slice of the frontal lobe was collected and frozen for biochemical studies. The remaining brain was used for histological analysis.

### Neurological examination

Neurological assessments were adapted from a previous study of the GM1 mouse model.^18^ These assessments were selected to reflect neurological signs characteristic of this model. A blinded examiner evaluated nine different parameters: gait, forelimb position, hindlimb position, trunk position, tail position, avoidance response, rolling over, vertical righting reflex, and parachute reflex. Individual test items were assigned one of the following four scores: 0 (normal), 1 (slightly abnormal), 2 (moderately abnormal), and 3 (highly abnormal). Scores for each parameter were added to calculate a total score.

### Vector

Vectors were constructed from cis-plasmids containing the codon-optimized human *GLB1* coding sequence expressed from the chicken beta actin promoter with a cytomegalovirus enhancer (CB7), human elongation initiation factor 1 alpha promoter (EF1a), or human ubiquitin C promoter (UbC) flanked by AAV2 inverted terminal repeats. The vectors were packaged in an AAV serotype hu68 capsid by triple transfection of adherent HEK 293 cells and purified by iodixanol gradient centrifugation as previously described^19^ for the pilot expression study and by affinity chromatography followed by anion exchange chromatography for the *Glb1* knockout mouse study.

### Enzyme activity assays

Tissues were homogenized in 0.9% NaCl, pH 4.0 by using a steel bead homogenizer (TissueLyzer; Qiagen). After three freeze-thaw cycles, samples were clarified by centrifugation and protein content was quantified by a bicinchoninic acid assay. Serum samples were used directly for enzyme assays. For the β-gal activity assay, a 1 μL sample was combined with 99 μL of 0.5 mM 4-methylumbelliferyl *β*-d-galactopyranoside (M1633; Sigma) in 0.15 M NaCl, 0.05% Triton-X100, 0.1 M sodium acetate, pH 3.58. The reaction was incubated at 37°C for 30 min and then stopped by addition of 150 μL of 290 mM glycine, 180 mM sodium citrate, pH 10.9.

Fluorescence was compared with standard dilutions of 4MU. β-gal activity is expressed as nmol 4MU liberated per hour per mg of protein (tissues) or per ml of serum or CSF. The HEX assay was performed in the same manner as the β-gal activity assay by using 1 mM 4-methylumbelliferyl *N*-acetyl-*β*-d-glucosaminide (M2133; Sigma) as a substrate and sample volumes of 1 μL for tissue lysates and 2 μL for serum.

### Histology

Brains were fixed overnight in 4% paraformaldehyde, equilibrated in 15% and 30% sucrose, and finally frozen in optimal cutting temperature embedding medium. Cryosections were stained with antibodies against lysosomal-associated membrane protein 1 (LAMP1) (Cat. No. Ab24170; Abcam) overnight at 4°C. The next day, slides were washed and incubated with an anti-rabbit IgG TritC-conjugated secondary antibody for 1 h at room temperature. Slides were washed, and coverslips were applied. LAMP1 staining was quantified as positive cells per field of the whole cerebral cortex from one coronal brain section by using VisioPharm image analysis software.

### Gait analysis

Motor function assessments were performed by using the CatWalk XT gait analysis system (Noldus Information Technology, Wageningen, The Netherlands). The CatWalk XT tracks the footprints of mice as they walk across a glass plate. The system quantifies the dimensions of each paw print and statistically analyzes the animal's speed and other features of gait.

To perform this assessment, the Catwalk XT was calibrated with the appropriate width of the walkway set before the start of the test. Animals were brought into the room and allowed to acclimate in darkness for at least 30 min before running the Catwalk XT. Once acclimation was complete, an animal was selected and placed at the entrance of the walkway. The researcher started the acquisition software and allowed the animal to walk down the walkway. The animal's home cage was placed at the end of the walkway for encouragement. The run was complete when the animal had successfully walked to the end of the catwalk within the allotted time limit; otherwise, the run was repeated.

Animals ran three trials with a minimum duration of 0.50 s and a maximum duration of 5.00 s. Three successful runs were needed for the trial to be considered complete. If an animal failed to complete three runs after 10 min of testing, only the completed runs were used for analysis. The analyses were performed by an evaluator blinded to the animal ID and treatment group. Runs were auto-classified by using the Catwalk XT software, after which footprints were checked for accuracy and proper labeling. Any nonfootprint data were manually removed. Average speed, stride length, and hind footprint length were automatically measured by the program. Mean values for the left and right hind paw print lengths were calculated and analyzed for each group. Mean values for stride length measured from each paw were calculated and analyzed for each group.

### Statistics

Analyses were performed by using Prism 7.0 (GraphPad Software). Neurological exam scores and gait analysis parameters (walking speed and hind print length) were compared between groups at each time point by using a two-way analysis of variance (ANOVA). Survival curves were compared between groups by using a log-rank (Mantel-Cox) test. Brain LAMP1 data were log-transformed and compared by using a one-way ANOVA followed by Dunnett's test.

## Results

We selected the AAVhu68 capsid based on previous studies demonstrating that clade F AAV isolates are capable of efficient gene transfer to the brain and spinal cord after delivery into the CSF.^13^ We designed transgene cassettes consisting of a codon-optimized human *GLB1* cDNA driven by one of three ubiquitous promoters: chicken beta actin with a cytomegalovirus enhancer (CB7), elongation factor 1 alpha (EF1α) or ubiquitin C (UbC). We packaged each cassette in an AAVhu68 capsid, and we administered a single dose of 10^11^ genome copies (GCs) to wild-type mice by ICV injection. Two weeks after injection, we measured β-gal activity in brain and CSF (Supplementary Fig. S1). Only the vector carrying the UbC promoter achieved statistically significant elevations in β-gal activity in both the brain and CSF, with enzyme activity nearly twofold greater than that of untreated wild-type mice in the brain, and tenfold greater in CSF. We, therefore, selected the AAVhu68.UbC.hGLB1 vector for further studies.

We assessed the efficacy of the optimized vector in the Glb1^−/−^ mouse model. This mouse model of GM1 gangliosidosis was developed by targeted deletion of exon 15 of the *Glb1* gene.^20,21^ Similar to infantile GM1 gangliosidosis patients, these mice express no functional β-gal and exhibit rapid accumulation of GM1 ganglioside in the brain. Brain GM1 storage is already apparent in the first weeks of life, and by 3 months of age, *Glb1^−/−^* mice have a similar degree of GM1 accumulation in the brain to that of an 8-month-old infantile GM1 patient.^20^

The clinical phenotype of the *Glb1^−/−^* mouse most closely resembles that of infantile GM1 gangliosidosis, with motor abnormalities appearing by 4 months of age and severe ataxia or paralysis necessitating euthanasia presenting by 10 months of age.^20,21^ The *Glb1^−/−^* mouse model does not exhibit any peripheral organ involvement, unlike infantile GM1 patients who often develop bone deformities and hepatosplenomegaly.^20,21^ The *Glb1^−/−^* mouse is, therefore, a representative model of the neurological features of infantile GM1 gangliosidosis, but not the systemic disease manifestations.

One-month-old *Glb1^–/–^* mice received a single ICV administration of AAVhu68.UbC.hGLB1 at one of four doses: 4.4 × 10^9^ GC, 1.3 × 10^10^ GC, 4.4 × 10^10^ GC, or 1.3 × 10^11^ GC. Control *Glb1^–/–^* mice and normal *Glb1^+/–^* mice were administered vehicle (artificial CSF) ICV. In-life assessments included monitoring for survival, neurological exams, gait analysis, and evaluation of serum transgene expression (β-gal activity). Necropsies were performed on the day of dosing (day 1) for untreated *Glb1^–/–^* mice and normal *Glb1^+/–^* mice to evaluate the severity of baseline brain storage lesions. Vehicle- and vector-treated mice were necropsied on day 150 and 300.

**Figure 1.**
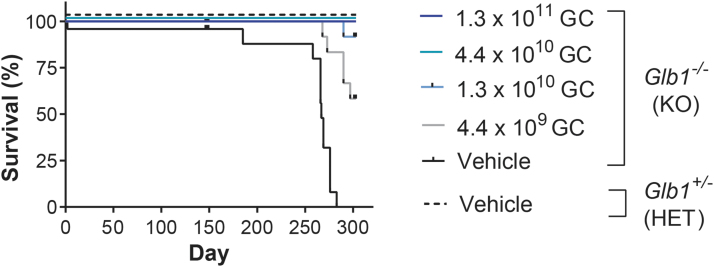
Survival of *Glb1^–/–^* mice treated with vector or vehicle. *Glb1^–/–^* (KO) mice were ICV-administered AAVhu68.UbC.hGLB1 at a dose of 1.3 × 10^11^ GC, 4.4 × 10^10^ GC, 1.3 × 10^10^ GC, or 4.4 × 10^9^ GC (*N* = 12/group). *Glb1^–/–^* (KO) and *Glb1*^+/–^ (HET) mice were ICV-administered vehicle as controls (*N* = 12/group). Survival was monitored. Data for the day 150 and 300 cohorts are combined by treatment and genotype. GC, genome copies; HET, heterozygote; ICV, intracerebroventricular; KO, knockout.

In the day 150 cohort, all mice survived to the scheduled necropsy except one vehicle-treated *Glb1^–/–^* mouse (Fig. 1). This animal died 2 days after vehicle administration due to intracranial hemorrhage, likely caused by the ICV injection procedure.

In the day 300 cohort, all 12 vehicle-treated *Glb1^–/–^* mice were euthanized according to the study-defined euthanasia criteria before the scheduled study endpoint. The mice exhibited neurological signs (*i.e.*, ataxia, tremors, and/or limb weakness) as a result of disease progression. The median survival of vehicle-treated *Glb1^–/–^* mice was 268 days (ranging from 185 to 283 days). In the lowest dose group (4.4 × 10^9^ GC), 5 out of 12 (41.7%) animals were euthanized due to disease progression, with survival ranging from 268 to 297 days. A single animal (1/12 [8.3%]) in the 1.3 × 10^10^ GC dose cohort was euthanized due to disease progression 290 days post-treatment. All animals that received vector doses of 4.4 × 10^10^ GC or 1.3 × 10^11^ GC survived to the study endpoint.

Gait analysis evaluated the stride length and hind paw print length of vehicle- and vector-treated mice at baseline (day −7 to 0) and every 60 days through day 240. Gait analysis revealed progressive abnormalities in vehicle-treated *Glb1^–/–^* mice, whereas *Glb1^–/–^* mice treated with the two highest vector doses (1.3 × 10^11^ GC and 4.4 × 10^10^ GC) demonstrated consistent improvements in both gait parameters.

**Figure 2.**
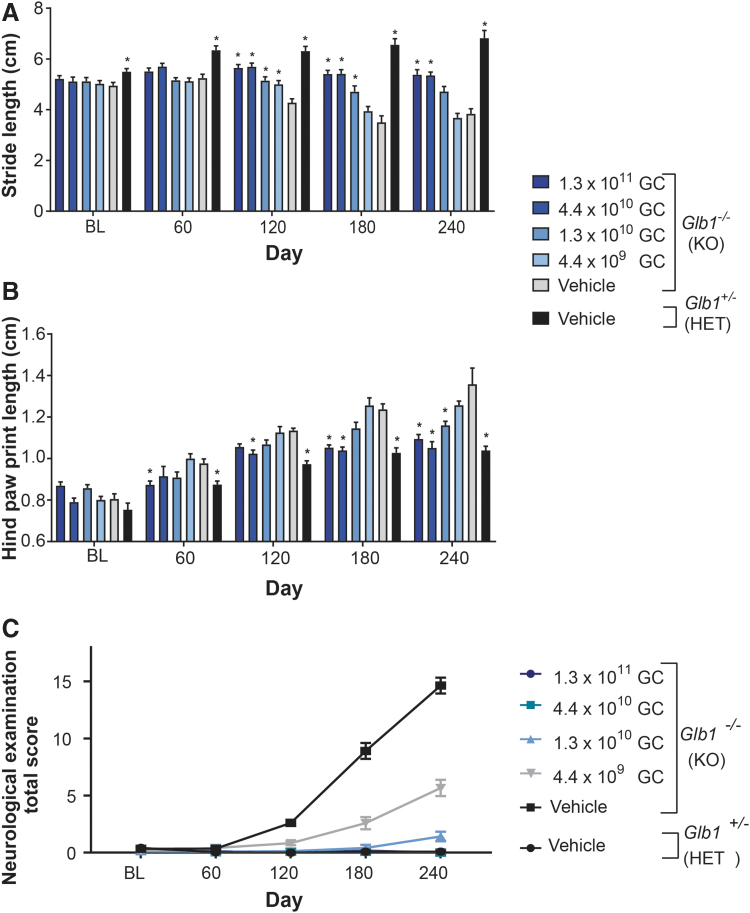
Gait and neurological assessment of *Glb1^–/–^* mice treated with vector or vehicle. *Glb1^–/–^* (KO) mice were ICV-administered AAVhu68.UbC.hGLB1 at a dose of 1.3 × 10^11^ GC, 4.4 × 10^10^ GC, 1.3 × 10^10^ GC, or 4.4 × 10^9^ GC (*N* = 12/group). *Glb1^–/–^* (KO) and *Glb1*^+/–^ (HET) mice were ICV-administered vehicle as controls (*N* = 12/group). Gait assessment was performed at baseline (days −7 to 0) and on days 60 ± 3, 120 ± 7, 180 ± 7, and 240 ± 7 by using the CatWalk XT system. Data for the day 150 and 300 cohorts are combined by treatment and genotype, and average stride length **(A)** and average length of the hind paw prints **(B)** are presented for each time point. Error bars represent the SEM. *p < 0.05 versus vehicle-treated *Glb1^–/–^* (KO) controls based on a two-way ANOVA followed by Dunnett's test. **(C)** Standardized neurological examinations were performed at BL (days −7 to 0) and on days 60 ± 3, 120 ± 7, 180 ± 7, and 240 ± 7. All vector-treated groups demonstrated significant reductions in neurological exam scores compared with vehicle-treated *Glb1^–/–^* (KO) controls on days 120 ± 7, 180 ± 7, and 240 ± 7 (*p* < 0.001 based on a two-way ANOVA followed by Tukey's multiple-comparisons test). ANOVA, analysis of variance; BL, baseline; SEM, standard error of the mean.

At baseline, the average stride length (Fig. 2A) of vehicle-treated *Glb1^–/–^* mice was significantly shorter than that of normal *Glb1^+/–^* controls, and this abnormality persisted through day 240. The stride length abnormality was partially rescued in vector-treated *Glb1^–/–^* mice, which displayed a statistically significant increase in average stride length compared with that of the vehicle-treated *Glb1^–/–^* mice at all doses by day 120. However, by day 240, only the two highest dose groups (1.3 × 10^11^ GC and 4.4 × 10^10^ GC) maintained a significantly longer average stride length compared with that of the vehicle-treated *Glb1^–/–^* mice.

On day 60, the average hind paw print length (Fig. 2B) of vehicle-treated *Glb1^–/–^* mice was significantly longer than that of normal *Glb1^+/–^* controls, and this abnormality persisted through day 240. The hind paw print length abnormality was partially rescued by vector administration in *Glb1^–/–^* mice at the three highest doses (1.3 × 10^11^ GC, 4.4 × 10^10^ GC, and 1.3 × 10^10^ GC), resulting in a statistically significant decrease in average hind paw print length compared with that of the vehicle-treated *Glb1^–/–^* mice by day 240.

A standardized neurological examination was performed in a blinded fashion every 60 days through day 240 (Fig. 2C). Beginning at the day 120 assessment, *Glb1^–/–^* mice administered either vehicle or the lowest dose of vector (4.4 × 10^9^ GC) exhibited progressively higher total severity scores, which was indicative of increasing severity of neurological signs. However, the total severity scores of the *Glb1^–/–^* mice administered the lowest dose were significantly lower than those of vehicle-treated *Glb1^–/–^* mice, suggesting that this dose (4.4 × 10^9^ GC) partially rescued the neurological phenotype. At the next highest dose (1.3 × 10^10^ GC), minimal abnormalities were detectable in 7 out of 12 (58.3%) animals at the day 240 assessment, suggesting substantial rescue of the neurological phenotype. At the two highest vector doses (1.3 × 10^11^ GC and 4.4 × 10^10^ GC), neurological abnormalities were not apparent, and total severity scores for these groups were similar to those of the normal vehicle-treated *Glb1^+/–^* controls at each time point, suggesting complete rescue of the neurological phenotype.

**Figure 3.**
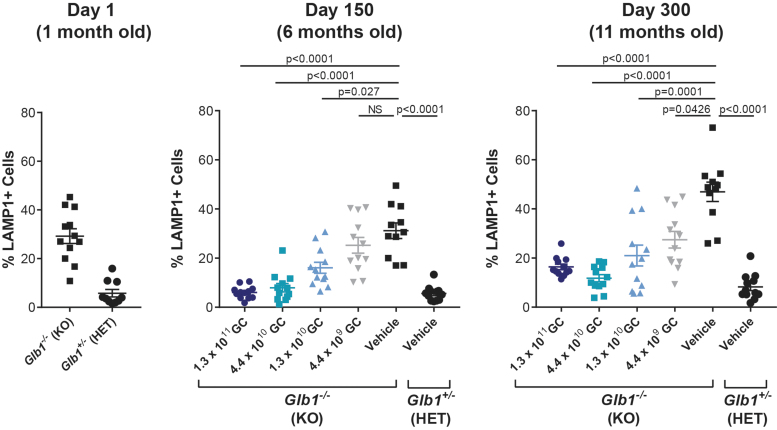
Lysosomal storage lesions in the brain of *Glb1^–/–^* mice treated with vector or vehicle. On days 150 ± 7 and 300 ± 7, brains were collected from *Glb1^–/–^* (KO) mice ICV-administered AAVhu68.UbC.hGLB1 at a dose of 1.3 × 10^11^ GC, 4.4 × 10^10^ GC, 1.3 × 10^10^ GC, or 4.4 × 10^9^ GC (*N* = 12/group) and from *Glb1^–/–^* (KO) and *Glb1*^+/–^ (HET) controls treated with ICV-administered vehicle (*N* = 12/group). On day 1 (baseline cohort), brains were also collected from untreated *Glb1^–/–^* (KO) and *Glb1*^+/–^ (HET) controls. Coronal brain sections were stained with an antibody against murine LAMP1. Automated quantification of LAMP1-positive cells in the entire cortex of one coronal brain section per animal was performed. For animals that did not survive to the scheduled day 300 necropsy due to disease progression, brains were collected at the time of euthanasia, and data are presented as part of the day 300 cohort. Significance was evaluated by a one-way ANOVA of log-transformed data followed by Dunnett's test. Error bars represent the SEM. LAMP1, lysosomal-associated membrane protein 1.

To evaluate the extent of lysosomal storage lesions, brain sections were stained for the lysosomal membrane protein LAMP1, and cortical cells positive for LAMP1 (*i.e.*, cells exhibiting lysosomal distention) were quantified in scanned sections by using an automated program. Untreated *Glb1^–/–^* baseline control mice necropsied on day 1 exhibited a higher proportion of LAMP1-positive cells in the brain compared with that of normal untreated *Glb1^+/–^* baseline controls. At both day 150 and 300, vector-treated animals exhibited a dose-dependent reduction in the proportion of LAMP1-positive cells compared with that of vehicle-treated *Glb1^–/–^* controls (Fig. 3). Of note, the two highest vector doses (1.3 × 10^11^ GC and 4.4 × 10^10^ GC) reduced the proportion of LAMP1-positive cells to levels similar to those of normal vehicle-treated *Glb1^+/–^* controls. Correction of lysosomal storage was apparent throughout the coronal brain sections, reflecting treatment of both the superficial and deep brain structures.

**Figure 4.**
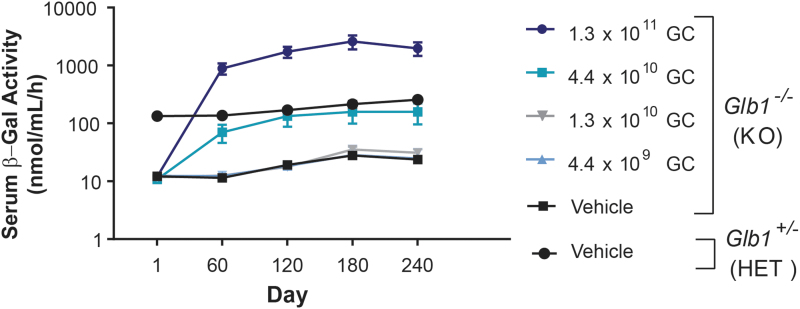
Beta-galactosidase activity in serum of *Glb1^–/–^* mice treated with vector or vehicle. *Glb1^–/–^* (KO) mice were ICV-administered AAVhu68.UbC.hGLB1 at a dose of 1.3 × 10^11^ GC, 4.4 × 10^10^ GC, 1.3 × 10^10^ GC, or 4.4 × 10^9^ GC (*N* = 12/group). *Glb1^–/–^* (KO) and *Glb1*^+/–^ (HET) mice were ICV-administered vehicle as controls (*N* = 12/group). Serum was collected on the day of dosing (day 1) and on days 60 ± 3, 120 ± 7, 180 ± 7, and 240 ± 7. Serum β-gal activity was measured by using a fluorogenic substrate. Data for the day 150 and 300 cohorts are combined by treatment and genotype. Error bars represent the SEM. β-gal, beta-galactosidase.

β-gal activity was measured in serum on the day of dosing (day 1) and every 60 days thereafter until day 240 (Fig. 4). At necropsy, β-gal activity was measured in the brain and peripheral organs (heart, liver, spleen, lung, and kidney). In serum, average β-gal activity in *Glb1^–/–^* mice administered the highest dose of vector (1.3 × 10^11^ GC) was ∼10-fold greater than that of normal vehicle-treated *Glb1^+/–^* controls. At the second highest dose (4.4 × 10^10^ GC), serum β-gal activity in *Glb1^–/–^* mice was similar to that of normal vehicle-treated *Glb1^+/–^* controls. Serum β-gal activity in *Glb1^–/–^* mice for all other vector doses was similar to that of vehicle-treated *Glb1^–/–^* controls (Fig. 4).

**Figure 5.**
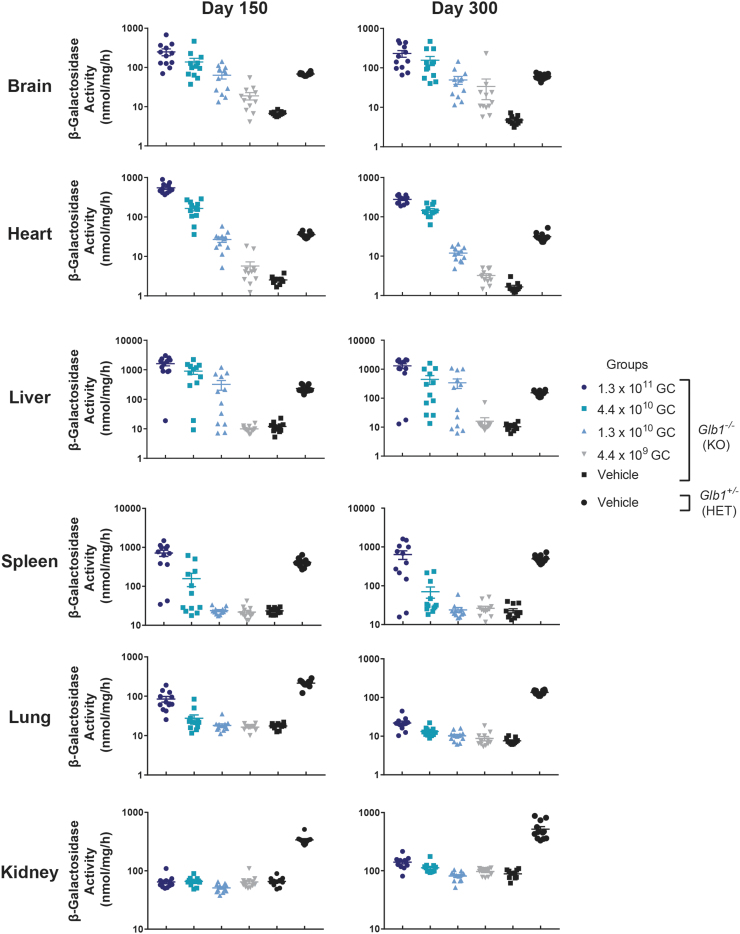
β-gal activity in tissues of *Glb1^–/–^* mice treated with vector or vehicle. *Glb1^–/–^* (KO) mice were ICV-administered AAVhu68.UbC.hGLB1 at a dose of 1.3 × 10^11^ GC, 4.4 × 10^10^ GC, 1.3 × 10^10^ GC, or 4.4 × 10^9^ GC (*N* = 12/group). *Glb1^–/–^* (KO) and *Glb1*^+/–^ (HET) mice were ICV-administered vehicle as controls (*N* = 12/group). Brain, heart, lung, liver, spleen, and kidney were collected at necropsy on days 150 ± 7 or 300 ± 7. β-gal activity was measured by using a fluorogenic substrate. For animals that did not survive to the scheduled day 300 necropsy due to disease progression, tissues were collected at the time of euthanasia, and data are presented as part of the day 300 cohort. Error bars represent the SEM.

For each tissue type examined, average β-gal activity levels within each group were similar at both time points (day 150 and 300) (Fig. 5). In the brain, β-gal activity increased in a dose-dependent manner in vector-treated *Glb1^–/–^* mice. Average β-gal activity for all dose groups was higher than that of the vehicle-treated *Glb1^–/–^* controls. However, only the two highest dose groups (1.3 × 10^11^ GC and 4.4 × 10^10^ GC) exhibited higher average β-gal activity than that of the normal vehicle-treated *Glb1^+/–^* controls at both time points. Some peripheral organs (*e.g.*, liver and spleen) but not all (*e.g.*, lung and kidney) exhibited increases in β-gal activity after vector administration (Fig. 5). Of particular note, the heart displayed dose-dependent increases in β-gal activity, resulting in average levels higher than those of vehicle-treated *Glb1^–/–^* mice at all doses. However, only the two highest doses (1.3 × 10^11^ GC and 4.4 × 10^10^ GC) restored β-gal activity to levels similar to or higher than those of normal vehicle-treated *Glb1^+/–^* controls at both time points.

**Figure 6.**
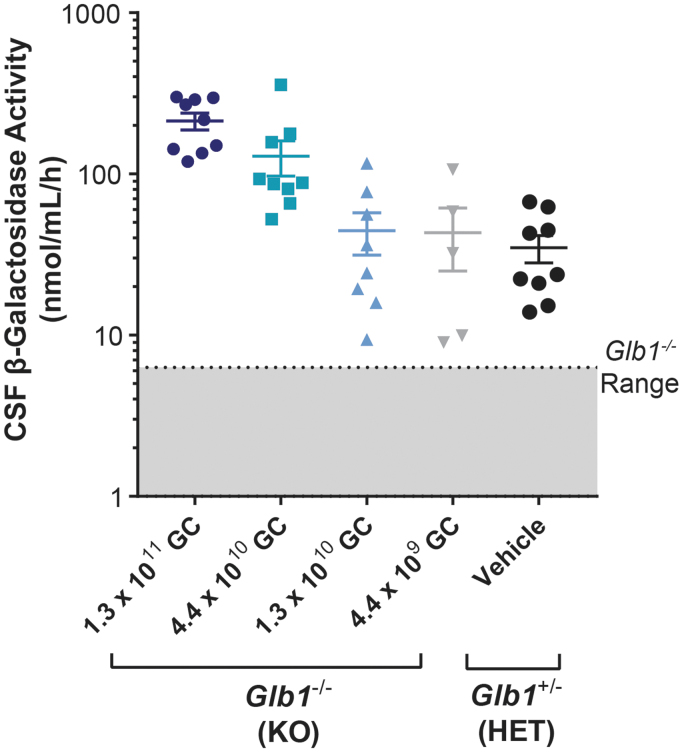
β-gal activity in CSF of vector-treated *Glb1^–/–^* mice and vehicle-treated controls. CSF was collected from *Glb1^–/–^* (KO) mice that survived to day 300 after ICV administration of AAVhu68.UbC.hGLB1 at a dose of 1.3 × 10^11^ GC (*N* = 9), 4.4 × 10^10^ GC (*N* = 9), 1.3 × 10^10^ GC (*N* = 8), or 4.4 × 10^9^ GC (*N* = 5). CSF was also collected from *Glb1*^+/–^ (HET) controls after ICV administration of vehicle (*N* = 9). β-gal activity was measured in CSF by using a fluorogenic substrate. CSF was not collected from vehicle-treated *Glb1^–/–^* (KO) mice, because none survived to day 300. The range of β-gal activity in CSF from *Glb1^–/–^* mice is based on data from 10 vehicle-treated animals from a prior study (*shaded area*). Error bars represent the SEM. CSF, cerebrospinal fluid.

β-gal activity was measured in the CSF of all animals in the day 300 cohort that survived to the scheduled necropsy. Because none of the vehicle-treated *Glb1^–/–^* animals survived to day 300 due to disease progression, β-gal activity levels of vector-treated mice were compared with those of normal vehicle-treated *Glb1^+/–^* controls (Fig. 6).

β-gal activity was detectable in the CSF of all mice evaluated. *Glb1^–/–^* mice administered the two highest doses (1.3 × 10^11^ GC and 4.4 × 10^10^ GC) displayed average CSF β-gal activity levels exceeding those of normal vehicle-treated *Glb1^+/–^* controls. β-gal activity in CSF was generally dose dependent, whereas β-gal activity in the two lowest dose groups appeared to be similar (1.3 × 10^10^ GC and 4.4 × 10^9^ GC) to that of the vehicle-treated *Glb1^+/–^* controls.

The reason for similar β-gal activity levels at the two lowest doses might be related to the number of CSF samples from the animals administered the lowest vector dose (4.4 × 10^9^ GC), which was limited by this group's high mortality; the animals that survived in this group might have had higher β-gal expression than the others that did not. In all groups, the levels of β-gal activity exceeded the range observed for CSF from historical control vehicle-treated *Glb1^–/–^* mice (gray area).

## Discussion

More than 40 years after researchers identified the underlying biochemical defect of GM1 gangliosidosis, there remain no disease-modifying therapies for this disease. For other neurodegenerative lysosomal storage diseases, hematopoietic stem cell transplantation (HSCT) has been successfully employed to deliver the deficient enzyme to the CNS via donor-derived cells that migrate into the brain. HSCT has been attempted in one presymptomatic GM1 gangliosidosis patient and in murine and canine models of GM1 gangliosidosis, with no impact on the neurodegenerative course of the disease.^22–24^ In contrast, mice transplanted with hematopoietic stem cells engineered to overexpress β-gal exhibited improvements in neuronal storage lesions and neurologic signs, suggesting that HSCT alone is ineffective due to insufficient enzyme delivery.^22^

Studies have explored AAV-mediated gene transfer as an alternative method to rapidly achieve robust, durable β-gal expression in the CNS. Remarkable efficacy has been demonstrated after multiple intraparenchymal AAV injections in both murine and feline disease models.^4,5^ However, translating this approach to patients is complicated by the invasiveness of the injection procedure as well as the challenge of scaling the number of injections to achieve similar vector distribution in the larger human brain.

This study demonstrates the potential for delivery of an optimized vector into CSF to restore β-gal activity and correct storage lesions throughout the brain. Near-complete correction of the disease phenotype was achieved at a dose of 4.4 × 10^10^ GC (1.1 × 10^11^ GC/g brain mass), similar to doses employed in ongoing clinical trials for other lysosomal storage diseases (NCT03566043). A single AAV injection into the CSF of the cisterna magna results in diffuse CNS gene transfer in large animals, supporting direct translation of a similar approach to clinical studies in humans.^7–9,11–13,15^

In addition to neurological symptoms, GM1 gangliosidosis patients exhibit variable somatic disease.^1^ AAV delivery into the CSF results in systemic vector distribution and may achieve sufficient peripheral enzyme expression to attenuate skeletal dysplasia, cardiomyopathy, and hepatosplenomegaly.^8,12,13^ Pre-existing neutralizing antibodies to the vector capsid do not impact gene transfer to the brain, but may prevent peripheral transduction, potentially leading to different degrees of peripheral β-gal expression in patients depending on neutralizing antibody status.^11,12^

This study demonstrated an absence of neuronal storage lesions in *Glb1^−/−^* mice treated with an AAV vector at 4 weeks of age, when prominent brain storage lesions are already present in this model.^20^ These results suggest that gene transfer may both prevent and reverse GM1 storage in the brain. However, GM1 storage ultimately results in neuron death, meaning that it will be critical to identify and treat patients before irreversible neurodegeneration has occurred. Clinical trials of gene therapy for GM1 gangliosidosis will, therefore, face the challenge of identifying patients who are likely to develop neurological disease before symptoms manifest. Due to the high frequency of private mutations, prediction of clinical phenotype is usually not possible based on genotype alone.^1,2^

Patients with infantile GM1 gangliosidosis may be an ideal population to evaluate AAV gene therapy, as they are frequently diagnosed based on subtle neurological findings that appear in the first 6 months of life before the onset of the rapid developmental regression that inevitably follows within 1–2 years.^3^

## Supplementary Material

Supplemental data
