# Supplementary material for: A Single Injection of an Optimized Adeno-Associated Viral Vector into Cerebrospinal Fluid Corrects Neurological Disease in a Murine Model of GM1 Gangliosidosis
Source: Hum Gene Ther. 2020 Nov 13;31(21-22):1169–77. doi: 10.1089/hum.2018.206 (PMC7698982; doi:10.1089/hum.2018.206)
Supplement: Supplemental data [file Supp_Fig1.docx]

**
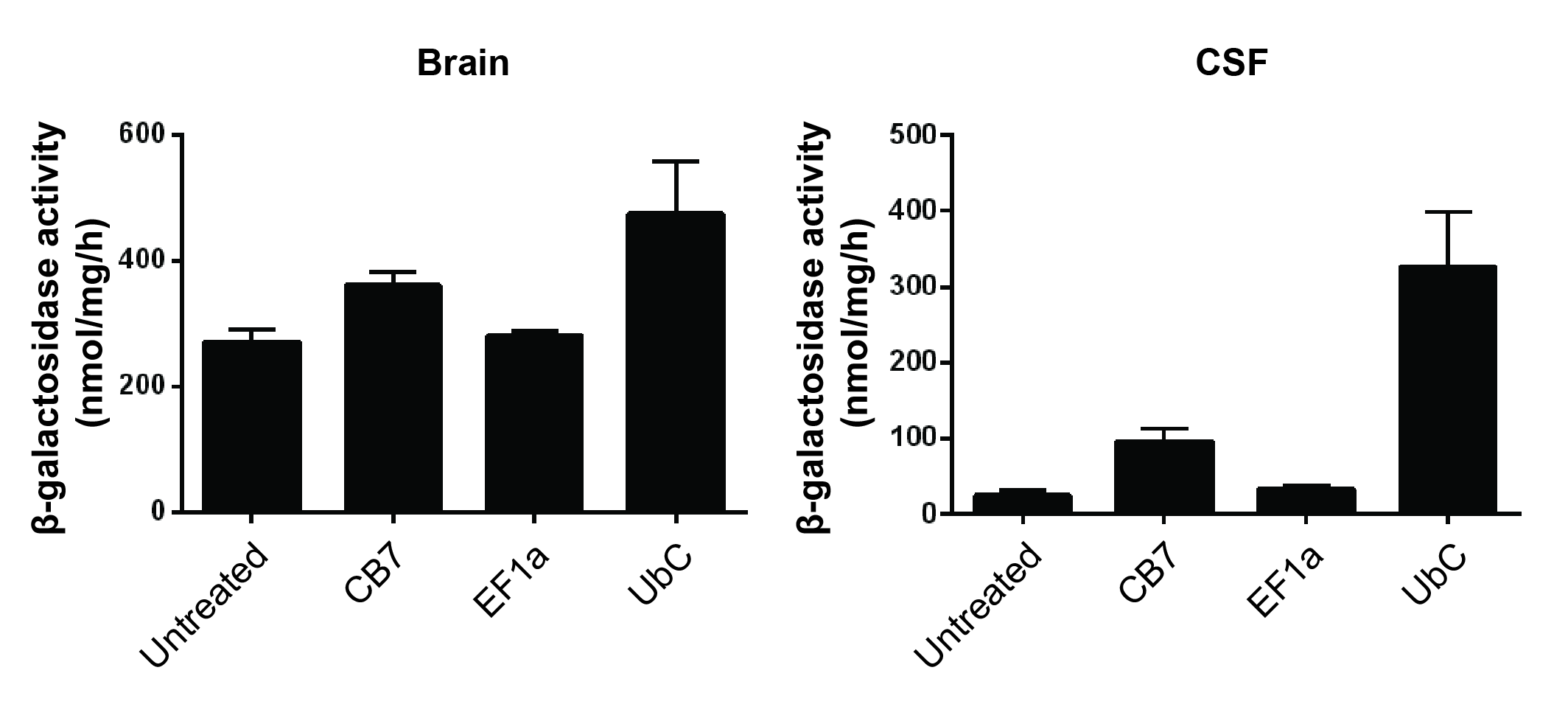
**

**Figure S1: β-gal activity in brain and CSF of wild-type mice treated with AAV vectors expressing human GLB1 using different promoters**

Wild-type mice were treated with a single ICV injection of AAVhu68 vectors expressing human GLB1 from a CB7, EF1a, or UbC promoter (n = 10 per group). Untreated wild-type mice (n = 5) served as controls. Brain (frontal cortex) and CSF were collected 14 days after vector administration, and β-gal activity was measured using a fluorogenic substrate. *p < 0.05, **p<0.01, ***p<0.001 compared to untreated, Kruskal-Wallis test followed by Dunn’s test. Error bars represent SEM.
